# Efficacy of nerve growth factor antibody in a knee osteoarthritis pain model in mice

**DOI:** 10.1186/s12891-017-1792-x

**Published:** 2017-11-03

**Authors:** Masayuki Miyagi, Tetsuhiro Ishikawa, Hiroto Kamoda, Miyako Suzuki, Gen Inoue, Yoshihiro Sakuma, Yasuhiro Oikawa, Sumihisa Orita, Kentaro Uchida, Kazuhisa Takahashi, Masashi Takaso, Seiji Ohtori

**Affiliations:** 1 Department of Orthopaedic Surgery, Kitasato University, School of Medicine, 1-15-1, Kitasato, Minami-ku, Sagamihara city, Kanagawa 252-0374 Japan; 2 Department of Orthopaedic Surgery, Graduate School of Medicine, Chiba University, Chiba, Japan

**Keywords:** Nerve growth factor, Anti-nerve growth factor therapy, Knee osteoarthritis, Monoiodoacetate, CatWalk, Calcitonin gene-related peptide, Dorsal root ganglia

## Abstract

**Background:**

Nerve growth factor (NGF) is not only an important factor in nerve growth but also a major contributor to the production of inflammation. It has been reported that inhibiting NGF could reduce several types of pain in several animal models. Here, we aimed to clarify the efficacy of NGF antibody in a knee osteoarthritis (OA) pain model in mice.

**Method:**

Six-week-old male C57BR/J mice were used (*n* = 30). Ten mice comprised the control group, which received saline injection into the right knee joints; the other 20 mice comprised the experimental group, which received monoiodoacetate (MIA) injection into the right knee joints. Three weeks after surgery, the 20 experimental mice were randomly placed into treatment groups which received either sterile saline (non-treat group: 10 mg/kg, i.p.) or an anti-NGF antibody (anti-NGF group: 10 mg/kg, i.p.). Simultaneously, all mice received fluorogold (FG) retrograde neurotracer injection into their right joints. In a behavioral study, we evaluated gait using the CatWalk quantitative gait analysis system before surgery, 3 weeks after surgery (before treatment), 4 weeks after surgery (one week after surgery), and 5 weeks after surgery (2 weeks after surgery). In immunohistochemical analysis, the right dorsal root ganglia (DRGs) from the L4–L6 levels were resected 5 weeks after surgery (2 weeks after surgery). They were immunostained for calcitonin gene-related peptide (CGRP), and the number of FG-labeled or CGRP-immunoreactive (IR) DRG neurons was counted.

**Results:**

On gait analysis using the CatWalk system, duty cycle, swing speed, and print area were decreased in non-treat group compared with those in control group and improved in the anti-NGF group compared with those in non-treat group. CGRP expression in DRGs was up-regulated in non-treat group compared with that in control group and suppressed in the anti-NGF group compared with that in non-treat group (both *p* < 0.05).

**Conclusions:**

MIA injection into the knee joint induced gait impairment and the up-regulation of CGRP in DRG neurons in a knee OA pain model in mice. Intraperitoneal injection of anti-NGF antibody suppressed this impairment of gait and up-regulation of CGRP in DRG neurons. These finding suggest that anti-NGF therapy might be valuable in the treatment of OA pain in the knee.

## Background

Knee osteoarthritis (OA) is a common chronic degenerative disease characterized by degeneration of articular cartilage components, synovitis, remodeling of subchondral bone and atrophy of joint muscles. Knee OA patients usually suffer from knee pain and are treated with several treatment modes, including medication, intraarticular injection of hyaluronic acid, and surgery [1]. Understanding of the mechanism of knee OA pain is incomplete, and medication for knee OA pain were sometimes insufficient.

As part of studies into new targets of knee OA pain, we have focused on pain-related molecules, including nerve growth factor (NGF) [2]. NGF not only plays an important role in the maintenance and development of the sensory nervous system [3] but is also a major contributor to inflammation and nociception [4]. Lewin et al. reported that the systemic injection of NGF induced thermal and mechanical hyperalgesia [5]. In addition, systemic injection of anti-NGF antibody reduced allodynia and hyperalgesia in animal models of neuropathic pain, including nerve trunk or spinal nerve ligation [6–8].

Basic research into knee OA has been aided by the development of several animal models, including the anterior cruciate ligament transection model [9], destabilization of the medial meniscus model [10], the rat medical meniscal tear model [11], GDF5 deficiency mice [12], and monoiodoacetate (MIA) injection model [13–16]. The MIA injection model has been reported to result in progressive joint damage, with some features that may be considered similar to OA [14–16] and significant pain-related behavior [13, 16]. The MIA injection model is superior for evaluating knee pain; although it seems animal models including the medical meniscus model better approximate the anatomic pathology found in OA in humans. Some of these previous reports evaluated pain-related behavior using the von Frey test, which is for evaluation of mechanical allodynia. Clinically, however, knee OA patients suffer from knee pain that includes gait impairment but not mechanical allodynia. Several authors have evaluated knee pain behavior using weight bearing assays [17–20]. For instance, the CatWalk gait analysis system can provide quantitative assessment of gait and motor function in rats and mice. This system has recently been used to assay impaired gait function in knee OA pain models [17–19]. Regarding the pathological mechanism of knee OA pain, our previous immunohistochemical analysis showed that the expression of pain-related molecules in the sensory nervous system was increased in a knee OA pain model [16]. Thus, this finding demonstrated the up-regulation of pain-related molecules in the sensory nervous system in the pain state.

We therefore hypothesized that anti-NGF therapy was effective for knee pain in a mouse model of knee OA. The aim of the current study is to evaluate the efficacy of NGF antibody in a knee OA pain model using the CatWalk gait analysis system and immunohistochemical analysis of the sensory nervous system in mice.

## Method

### Knee osteoarthritis pain model

Thirty 8-week-old male C57 BL/6 mice were used. (Control group: *n* = 10, Monoiodeacete (MIA) without treatment (non-treat) group: *n* = 10, and MIA + anti-NGF therapy (anti-NGF) group: *n* = 10) Animals were anesthetized with sodium pentobarbital (40 mg/kg, intraperitoneal) and treated aseptically throughout the experiments. In the non-treatment group and anti-NGF group, the right knees were treated with a single intraarticular injection of 0.2 mg of MIA (Sigma-Aldrich, St. Louis, MO) in 10 μl of sterile saline. The solution was injected through the patellar ligament by using a 27G needle with the leg flexed at a 90 degrees angle at the knee as described previously [16]. Three weeks after surgery, mice were randomly assigned to treatment groups receiving either sterile saline (10 mg/kg, i.p.) (non-treat group) or an anti-NGF antibody (L148 M; Exalpha Biological Inc., Shirley, MA) (10 mg/kg, i.p.) (anti-NGF group). We have previously confirmed this medication’s efficacy for treating neuropathic pain [2]. Simultaneously, to detect dorsal root ganglia (DRG) neurons innervating the right knee joint, the right knees of all 20 animals were treated with intraarticular injection of 2% retrograde neurotracer FG (Fluorochrome, Denver, CO) as we have previously described [16]. The control group was treated with a single intraarticular injection of 10 μl of sterile saline to the right knee, followed three weeks later by intraarticular injection of 2% retrograde neurotracer FG.

### Behavioral evaluation (gait analysis)

Gait was analysed in detail using the CatWalk system (Noldus Information Technology, Wageningen, The Netherlands). This system has been described in detail elsewhere [19]. Briefly, mice are placed on a glass plate located in a darkened room and allowed to walk freely. A light beam from a fluorescent lamp is aimed through the glass plate. The light beams are completely reflected internally. However, when a paw touches the glass plate, the light beams are reflected downwards. This results in a sharp bright image of the paw print. The entire run is recorded with a video camera. The data are acquired, compressed, and analyzed using CatWalk software as described previously [21].

Before surgery and 3 (before treatment; pre-treat) 4, or 5 weeks (1 or 2 weeks after treatment, respectively; treat-1w or treat-2w) after surgery, all mice were walked on the glass plate three times. The gait of all mice was recorded three times and analyzed using the CatWalk system. We compared the ratio of movement of the ipsi and contralateral hind paws with regard to three variables, namely duty cycle (standing as a percentage of the step cycle: stand/(stand time + swing time) × 100%); swing speed (speed of the paw during swing: stride length/swing time); and print area (surface area of the complete print) among the three groups.

### Immunohistochemical analysis

All procedure, including anesthetization, perfusion, sectioning, immunostaining and the observation and evaluation of immunoreactive neurons, were used the same manner as discribed previously [21, 22].

In all three groups, four (*n* = 5, each) or five (*n* = 5, each) weeks after surgery (one or two weeks after treatment), mice were anesthetized with sodium pentobarbital (40 mg/kg, intraperitoneal) and perfused transcardially with 0.9% saline, followed by 30 mL of 4% paraformaldehyde in phosphate buffer (0.1 M, pH 7.4). Next, the right DRGs from the L3 to L5 levels were resected and the specimens were immersed in the same fixative solution overnight at 4 °C, and then stored in 0.01 M phosphate-buffered saline (PBS) containing 20% sucrose for 20 h at 4 °C.

For DRGs, each ganglion was sectioned at a 10 μm thickness on a cryostat and mounted on POLY-L-LYSINE-coated slides. Endogenous tissue peroxidase activity was quenched by soaking sections in 0.3% hydrogen peroxide solution in 0.01 M PBS for 30 min. Specimens were then treated for 90 min at room temperature in blocking solution consisting of 0.01 M PBS containing 0.3% Triton X-100 and 3% skim milk. To evaluate the expression of neuropeptides in DRGs, the sections were processed for CGRP immunohistochemistry using a rabbit antibody to CGRP (1:1000; Chemicon, Temecula, CA) diluted in blocking solution, and incubated for 20 h at 4 °C. To detect CGRP in the DRGs, sections were incubated for 3 h with goat antirabbit Alexa 488 fluorescent antibody conjugate (1:400; Molecular Probes, Eugene, OR).

Thirty sections in each group (two sections per each DRG (L3, L4, L5) in 5 animals) were examined using a fluorescence microscope. For each DRG section, we counted the number of all Fluoro-Gold-labeled neurons and the number of Fluoro-Gold-labeled and CGRP-immunoreactive (IR) neurons per 0.45 mm^2^ in 10 randomly-selected fields among 30 sections in each group at ×400 magnification using a counting grid, as we have previously reported [16]. The proportion of FG-labeled and CGRP-IR DRG neurons among all FG-labeled neurons was then calculated. All FG-labeled neurons indicated neurons innervating the right knee joint, while FG-labeled and CGRP-IR neurons indicated pain-related neurons innervating the right knee joint.

### Statistical analysis

Using the ratio of ipsi and contralateral hind paw values for the three CatWalk variables of duty cycle, swing speed, and print area, the proportion of FG-labeled and CGRP-IR DRG neurons among all FG-labeled DRG neurons was compared among the three groups using a non-repeated measures ANOVA with Bonferroni’s correction. A *p-*value less than 0.05 was considered statistically significant.

## Results

### Behavioral analysis

**Fig. 1:**
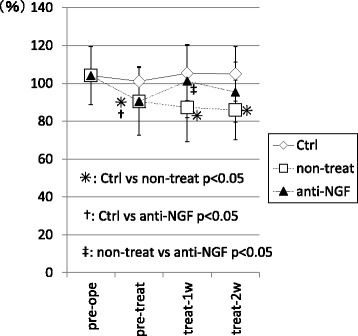
Ratio of ipsi and contralateral hind paw values of the duty cycle from the CatWalk system. (*p* < 0.05, compared among 3 groups using with a non-repeated measures ANOVA with Bonferroni’s *post-hoc* correction.). MIA injection of the knee joint induced a significant small duty cycle within 5 weeks after injection. In contrast, systemic injection of anti-NGF antibody induced significant improvement after only one week of treatment

In the non-treatment group, the ratio of ipsi to contralateral hind paw values for duty cycle was significantly decreased at pre-treat, treat-1w, and treat-2w compared with that in control group. (*p* < 0.05) In contrast, in the anti-NGF group, the ratio of ipsi to contralateral hind paws values for duty cycle were significantly decreased only at pre-treat (*p* < 0.05) compared with that in control group, and not at treat-1w and treat-2w (*p* > 0.05). In addition, the ratio in the anti-NGF group was significantly improved at treat-1w compared with that in the non-treat group. (*p* < 0.05) (Fig. 1).

**Fig. 2:**
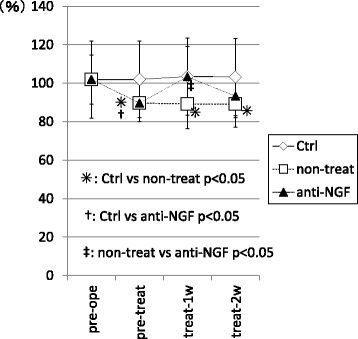
The ratio of ipsi and contralateral hind paw values for swing speed from the CatWalk system. (*p* < 0.05, compared among 3 groups using with a non-repeated measures ANOVA with Bonferroni’s *post-hoc* correction.). MIA injection of the knee joint induced a significant small swing speed within 5 weeks after injection. In contrast, systemic injection of anti-NGF antibody induced significant improvement after only one week of treatment

In the non-treated group, the ratio of ipsi to contralateral hind paw values for swing speed were significantly decreased at pre-treat, treat-1w, and treat-2w compared with that in the control group. (*p* < 0.05) In contrast, in the anti-NGF group, this ratio was significantly decreased only at pre-treat (*p* < 0.05) compared with that in control group, and not at treat-1w and treat-2w (*p* > 0.05). In addition, this ratio in the anti-NGF group was significantly improved at treat-1w compared with that in the non-treat group. (*p* < 0.05) (Fig. 2).

**Fig. 3:**
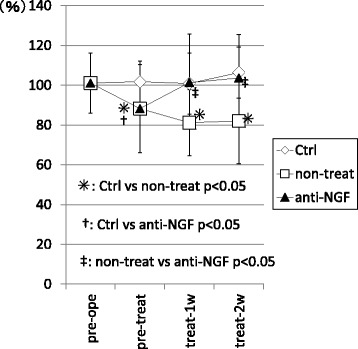
The ratio of ipsi and contralateral hind paw values of print area from the CatWalk system. (*p* < 0.05, compared among 3 groups using with a non-repeated measures ANOVA with Bonferroni’s *post-hoc* correction.). MIA injection of the knee joint induced a significant small print area within 5 weeks after injection. In contrast, systemic injection of anti-NGF antibody induced significant improvement after only 2 weeks of treatment

In the non-treated group, the ratio of ipsi and contralateral hind paw values for print area were significantly decreased at pre-treat, treat-1w, and treat-2w compared with that in control group. (*p* < 0.05) In contrast, in the anti-NGF group, this ratio was significantly decreased only at pre-treat (*p* < 0.05) compared with that in control group, and not at treat-1w and treat-2w (*p* > 0.05). In addition, the ratio in the anti-NGF group was significantly improved at treat-1w and treat-2w compared with that in the non-treat group. (*p* < 0.05) (Fig. 3).

### Immunohistochemical analysis

**Fig. 4:**
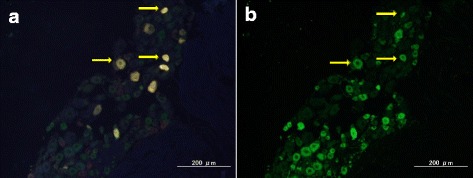
Representative FG-labeled (**a**) and CGRP-IR (**b**) DRG neurons. **a** and **b** are the same section. The arrow indicates FG- and CGRP-double labeled DRG neurons

FG-labeled DRG neurons, which indicated where FG was transported from the right knee, were present in the right L3 to L5 DRGs in all 3 groups (Fig. 4).

**Fig. 5:**
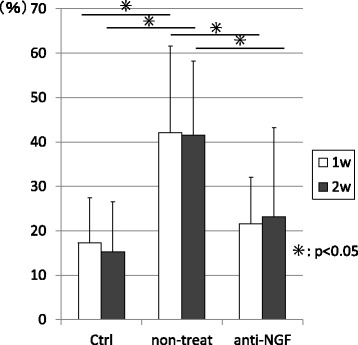
Percentages of FG and CGRP double labeling of DRGneurons among all FG labeled DRG neurons. (*p < 0.05, compared among the three groups using a non-repeated measures ANOVA with Bonferroni’s *post-hoc* correction)

The proportion of FG-labeled/CGRP-IR neurons among all FG-labeled neurons was significantly increased in the non-treated group at treat-1w and treat-2w compared with that in control group. (*p* < 0.05) Conversely, the expression of CGRP was significantly decreased in the anti-NGF group at treat-1w and treat-2w compared with the non-treated group (*p* < 0.05). (Figs. 4 and 5).

## Discussion

In this study, intraarticular injection of MIA to the knee joints induced gait disturbances, including a small duty cycle, small swing speed, and small print area, and the up-regulation of neuropeptides in DRG neurons innervating the knee joints. Systemic injection of anti-NGF antibody improved these gait disturbances and the expression of neuropeptides in DRG neurons in this model.

Regarding the Knee OA model, multiple authors have reported various surgery-induced animal models. Myers et al. reported that anterior cruciate ligament transection induced changes in the articular cartilage of the unstable knee that are consistent with OA [23]. In addition, Marijnissen et al. reported the usability of the “Groove” model, in which knee articular cartilage is damaged as a knee OA model [24]. Further, Bendele AM report that medial meniscal tear in rats results in rapidly progressive degenerative changes to the cartilage with similarities to OA [11]. These surgically induced knee OA models are suitable for basic research, to the extent that they simulate clinical situations. Nevertheless, the MIA injection model which results in severe joint damage seems superior for evaluating knee pain. MIA injection to the knee joints has been reported to cause joint pathology via the inhibition of glycolysis, and thereby targets avascular cartilage and causes chondrocyte death [25]. We also previously reported that MIA-treated knees exhibited behavioral disturbances within four days and osteoarthritic histological changes within four weeks [16]. We therefore used the MIA injection model as a knee OA model in this study.

Regarding knee pain behavior, several authors reported that knee pain models exhibited mechanical allodynia using the von Frey test [13, 16]. In addition, Shelton DL et al. report NGF induced mechanical allodynia in auto-immune arthritis [26]. Therefore, evaluating knee pain using the von Frey test seems to be appropriate for basic research using animal models. However, there is doubt about whether these models of knee pain simulate clinical situations, because knee OA patients do not typically complain of mechanical allodynia in their foot. Recently, gait analysis studies have focused on behavioral studies to evaluate pain behavior. The CatWalk system was initially developed to evaluate motor function, such as that modified in a spinal cord injury model, a Parkinson’s disease model, or an olivocerebellar degeneration model [19, 27–29]. Recently, this system has been applied to various models to evaluate behavior associated with pain, such as mechanical allodynia models [30]. We also previously evaluated low back pain behavior using the CatWalk system, and concluded that rat models of low back pain exhibit changes in rat gait, including a long standing time and short strides [21, 31]. Recently several authors applied the CatWalk system to evaluate behavior associated with knee pain. Ferland et al. reported that the MIA-induced knee pain model shows gait changes, including a short Duty cycle and a small Swing Speed. In our previous preliminary study, the MIA-induced knee pain model exhibited a small duty cycle, small swing speed, and small print area. (un-published date) Therefore, in this study, we compared the ratio of ipsi and contralateral hind paw values for the three variables, namely duty cycle, swing speed, and print area among the three groups to evaluate the effect of anti-NGF therapy.

Regarding the up-regulation of CGRP in the sensory nervous system, we previously evaluated potential pain-like states by evaluating the up-regulation of pain-related neuropeptides such as CGRP and substance P in the sensory nervous system using a combination of immunohistochemistry and retrograde tracing instead of evaluating pain behavior [22]. CGRP-IR DRG neurons have been reported to be NGF-dependent DRG neurons involved in pain perception related to inflammation [32], suggesting that CGRP is a marker of inflammatory pain. We previously reported that MIA injection to the knee joint induced the up-regulation of CGRP in DRGs [16]. These results suggest that the animals in the group receiving MIA injection to their knee joints were in a state of knee pain.

We focused on NGF as target for the treatment for knee OA pain based on our previous report that MIA injection induced the up-regulation not only inflammatory cytokines but also NGF in the knee joints [16]. Regarding the efficacy of anti-NGF therapy for knee OA pain, NGF is generally involved in chronic inflammatory or neuropathic pain states [33]. Several basic and clinical research had been reported the efficacy of anti-NGF therapy for knee OA pain [34, 35]. However, the pathological mechanism of knee OA pain was not fully understood. NGF is physically produced in articular structures and expressed in both normal and OA synovial tissues, but is increased in synovial inflammation, especially upon synovial tissue exposure to inflammatory cytokines including TNF-alpha [36, 37]. Thus, the up-regulation of inflammatory cytokines in OA knee joints promoted NGF production, which induced the up-regulation of CGRP in the sensory nervous system and chronic inflammatory or neuropathic pain states. Ashraf S et al. report that NGF injection into knee OA joints results in gait disturbance as compared to NGF injection into non-OA joints. This group used two models of knee OA, including an MIA injection model and a meniscal transection model [38]. Further, McNamee KE et al. report NGF expression is correlated with knee pain but that TNF alpha is not up-regulated in the late stages of OA [39]. These findings indicate inflammatory cytokines including TNF might only develop during the pain state, but that NGF might play a role in maintaining as well as in development of the pain state in knee OA patients. Therefore, NGF represents a possible important therapeutic target in the treatment of knee OA pain. In preclinical studies, newly developed tanezumab and its murine precursor muMab-911 have effectively targeted the NGF pathway in various chronic and inflammatory pain models [40]. In addition, phase I and II clinical trials for osteoarthritic pain and chronic lower back pain have demonstrated efficacy for the compound, as well as a good safety and tolerability profile [41]. In the present study, anti-NGF therapy suppressed the impairment gait and up-regulation of CGRP in DRG neurons. These findings may explain the pathological mechanism of knee OA pain.

There were some limitations in this study. First, we should have used tanezumab and its murine precursor muMab-911 for this study. We also did not evaluate differences in efficacy between muMab-911 and anti-NGF antibody, which we used in this study. Second, we did not evaluate the efficacy of positive control including non-steroid anti-inflammatory drugs for knee OA pain. Third, we also evaluated the efficacy of only a one-time injection of anti-NGF antibody. In the future, it will be necessary to evaluate the effect of repeated injections for knee pain. Fourth, we did not evaluate the histopathology of the knee joint. In clinical trials of the monoclonal NGF antibody tanezumab for knee and hip OA pain, osteonecrosis was reported as a side effect [42]. Therefore, further study focusing on side effects of anti-NGF therapy is necessary. Fifth, we did not include a power calculation to justify the group sizes. Further, due to ethical concerns related to using the animals, we combined our behavioral and immunohistochemical evaluations. Therefore, with regard to behavioral evaluation, some experiments ended after 4 weeks and others ended after 5 weeks.

## Conclusions

MIA injection into the knee joint induced gait impairment and the up-regulation of CGRP in DRG neurons in a knee OA pain model in mice. Further, intraperitoneal injection of anti-NGF antibody suppressed gait impairment and the up-regulation of CGRP in DRG neurons. These finding indicate that anti-NGF therapy might be valuable in the treatment of OA pain in the knee.

## Abbreviations

CGRP: Calcitonin gene related peptide
DRG: dorsal root ganglion
FG: FluoroGold
IR: immunoreactive
MIA: monoiodeacetate
NGF: nerve growth factor
OA: osteoarthritis

## References

[1] Vaishya R, Pariyo GB, Agarwal AK, Vijay V (2016). Non-operative management of osteoarthritis of the knee joint. J Clin. Orthop Trauma.

[2] Miyagi M, Ishikawa T, Kamoda H, Suzuki M, Inoue G, Sakuma Y, Oikawa Y, Uchida K, Suzuki T, Takahashi K, Takaso M, Ohtori S. The efficacy of nerve growth factor antibody in a mouse model of neuropathic cancer pain. Exp.Anim 2016; 65 (4):337–343. 10.1538/expanim.16-0014 PMC5111836 27194075

[3] Mendell LM (1996). Neurotrophins and sensory neurons: role in development, maintenance and injury. A thematic summary. Philos.Trans.R.Soc.Lond B Biol.Sci.

[4] Lewin GR, Mendell LM (1993). Nerve growth factor and nociception. Trends Neurosci.

[5] Lewin GR, Rueff A, Mendell LM (1994). Peripheral and central mechanisms of NGF-induced hyperalgesia. Eur.J Neurosci.

[6] Ramer MS, Bisby MA (1999). Adrenergic innervation of rat sensory ganglia following proximal or distal painful sciatic neuropathy: distinct mechanisms revealed by anti-NGF treatment. Eur. J Neurosci.

[7] Ro LS, Chen ST, Tang LM, Jacobs JM (1999). Effect of NGF and anti-NGF on neuropathic pain in rats following chronic constriction injury of the sciatic nerve. Pain.

[8] Wild KD, Bian D, Zhu D, Davis J, Bannon AW, Zhang TJ, Louis JC (2007). Antibodies to nerve growth factor reverse established tactile allodynia in rodent models of neuropathic pain without tolerance. J Pharmacol.Exp.Ther.

[9] Ochiai N, Ohtori S, Sasho T, Nakagawa K, Takahashi K, Takahashi N, Murata R, Takahashi K, Moriya H, Wada Y, Saisu T (2007). Extracorporeal shock wave therapy improves motor dysfunction and pain originating from knee osteoarthritis in rats. Osteoarthritis. Cartilage..

[10] Muramatsu Y, Sasho T, Saito M, Yamaguchi S, Akagi R, Mukoyama S, Akatsu Y, Katsuragi J, Fukawa T, Endo J, Hoshi H, Yamamoto Y, Takahashi K (2014). Preventive effects of hyaluronan from deterioration of gait parameters in surgically induced mice osteoarthritic knee model. Osteoarthritis. Cartilage..

[11] Bendele AM (2001). Animal models of osteoarthritis. J Musculoskelet. Neuronal.Interact.

[12] Daans M, Luyten FP, Lories RJ (2011). GDF5 deficiency in mice is associated with instability-driven joint damage, gait and subchondral bone changes. Ann.Rheum.Dis.

[13] Combe R, Bramwell S, Field MJ (2004). The monosodium iodoacetate model of osteoarthritis: a model of chronic nociceptive pain in rats?. Neurosci.Lett.

[14] Guingamp C, Gegout-Pottie P, Philippe L, Terlain B, Netter P, Gillet P (1997). Mono-iodoacetate-induced experimental osteoarthritis: a dose-response study of loss of mobility, morphology, and biochemistry. Arthritis Rheum.

[15] Guzman RE, Evans MG, Bove S, Morenko B, Kilgore K (2003). Mono-iodoacetate-induced histologic changes in subchondral bone and articular cartilage of rat femorotibial joints: an animal model of osteoarthritis. Toxicol.Pathol.

[16] Orita S, Ishikawa T, Miyagi M, Ochiai N, Inoue G, Eguchi Y, Kamoda H, Arai G, Toyone T, Aoki Y, Kubo T, Takahashi K, Ohtori S (2011). Pain-related sensory innervation in monoiodoacetate-induced osteoarthritis in rat knees that gradually develops neuronal injury in addition to inflammatory pain. BMCMusculoskeletDisord.

[17] Ferland CE, Laverty S, Beaudry F, Vachon P (2011). Gait analysis and pain response of two rodent models of osteoarthritis. Pharmacol.Biochem.Behav.

[18] Ferreira-Gomes J, Adaes S, Castro-Lopes JM (2008). Assessment of movement-evoked pain in osteoarthritis by the knee-bend and CatWalk tests: a clinically relevant study. J Pain.

[19] Hamers FP, Lankhorst AJ, van Laar TJ, Veldhuis WB, Gispen WH (2001). Automated quantitative gait analysis during overground locomotion in the rat: its application to spinal cord contusion and transection injuries. J Neurotrauma.

[20] Nwosu LN, Mapp PI, Chapman V, Walsh DA (2016). Relationship between structural pathology and pain behaviour in a model of osteoarthritis (OA). Osteoarthritis. Cartilage..

[21] Miyagi M, Ishikawa T, Kamoda H, Orita S, Kuniyoshi K, Ochiai N, Kishida S, Nakamura J, Eguchi Y, Arai G, Suzuki M, Aoki Y, Toyone T, Takahashi K, Inoue G, Ohtori S (2011). Assessment of gait in a rat model of myofascial inflammation using the CatWalk system. Spine (Phila Pa 1976).

[22] Miyagi M, Ishikawa T, Orita S, Eguchi Y, Kamoda H, Arai G, Suzuki M, Inoue G, Aoki Y, Toyone T, Takahashi K, Ohtori S (2011). Disk injury in rats produces persistent increases in pain-related neuropeptides in dorsal root ganglia and spinal cord glia but only transient increases in inflammatory mediators: pathomechanism of chronic diskogenic low back pain. Spine (Phila Pa 1976).

[23] Myers SL, Brandt KD, O'Connor BL, Visco DM, Albrecht ME (1990). Synovitis and osteoarthritic changes in canine articular cartilage after anterior cruciate ligament transection. Effect of surgical hemostasis. Arthritis Rheum.

[24] Marijnissen AC, van Roermund PM, TeKoppele JM, Bijlsma JW, Lafeber FP (2002). The canine 'groove' model, compared with the ACLT model of osteoarthritis. Osteoarthritis. Cartilage..

[25] Janusz MJ, Bendele AM, Brown KK, Taiwo YO, Hsieh L, Heitmeyer SA (2002). Induction of osteoarthritis in the rat by surgical tear of the meniscus: inhibition of joint damage by a matrix metalloproteinase inhibitor. Osteoarthritis. Cartilage..

[26] Shelton DL, Zeller J, Ho WH, Pons J, Rosenthal A (2005). Nerve growth factor mediates hyperalgesia and cachexia in auto-immune arthritis. Pain.

[27] Cendelin J, Voller J, Vozeh F (2010). Ataxic gait analysis in a mouse model of the olivocerebellar degeneration. Behav. Brain Res.

[28] Chuang CS, Su HL, Cheng FC, Hsu SH, Chuang CF, Liu CS (2010). Quantitative evaluation of motor function before and after engraftment of dopaminergic neurons in a rat model of Parkinson's disease. J Biomed.Sci.

[29] Koopmans GC, Deumens R, Honig WM, Hamers FP, Steinbusch HW, Joosten EA (2005). The assessment of locomotor function in spinal cord injured rats: the importance of objective analysis of coordination. J Neurotrauma.

[30] Vrinten DH, Hamers FF (2003). CatWalk' automated quantitative gait analysis as a novel method to assess mechanical allodynia in the rat; a comparison with von Frey testing. Pain.

[31] Miyagi M, Ishikawa T, Kamoda H, Suzuki M, Sakuma Y, Orita S, Oikawa Y, Aoki Y, Toyone T, Takahashi K, Inoue G, Ohtori S (2013). Assessment of pain behavior in a rat model of intervertebral disc injury using the CatWalk gait analysis system. Spine (Phila Pa 1976).

[32] Averill S, McMahon SB, Clary DO, Reichardt LF, Priestley JV (1995). Immunocytochemical localization of trkA receptors in chemically identified subgroups of adult rat sensory neurons. Eur. J Neurosci.

[33] Pezet S, McMahon SB (2006). Neurotrophins: mediators and modulators of pain. Annu.Rev.Neurosci.

[34] Ishikawa G, Koya Y, Tanaka H, Nagakura Y (2015). Long-term analgesic effect of a single dose of anti-NGF antibody on pain during motion without notable suppression of joint edema and lesion in a rat model of osteoarthritis. Osteoarthritis. Cartilage..

[35] Schnitzer TJ, Marks JAA (2015). Systematic review of the efficacy and general safety of antibodies to NGF in the treatment of OA of the hip or knee. Osteoarthritis. Cartilage.

[36] Manni L, Lundeberg T, Fiorito S, Bonini S, Vigneti E, Aloe L (2003). Nerve growth factor release by human synovial fibroblasts prior to and following exposure to tumor necrosis factor-alpha, interleukin-1 beta and cholecystokinin-8: the possible role of NGF in the inflammatory response. Clin.Exp.Rheumatol.

[37] Takano S, Uchida K, Miyagi M, Inoue G, Fujimaki H, Aikawa J, Iwase D, Minatani A, Iwabuchi K, Takaso M (2016). Nerve growth factor regulation by TNF-alpha and IL-1beta in synovial macrophages and fibroblasts in osteoarthritic mice. J Immunol Res.

[38] Ashraf S, Mapp PI, Burston J, Bennett AJ, Chapman V, Walsh DA (2014). Augmented pain behavioural responses to intra-articular injection of nerve growth factor in two animal models of osteoarthritis. Ann.Rheum.Dis.

[39] McNamee KE, Burleigh A, Gompels LL, Feldmann M, Allen SJ, Williams RO, Dawbarn D, Vincent TL, Inglis JJ (2010). Treatment of murine osteoarthritis with TrkAd5 reveals a pivotal role for nerve growth factor in non-inflammatory joint pain. Pain.

[40] Xu L, Nwosu LN, Burston JJ, Millns PJ, Sagar DR, Mapp PI, Meesawatsom P, Li L, Bennett AJ, Walsh DA, Chapman V (2016). The anti-NGF antibody muMab 911 both prevents and reverses pain behaviour and subchondral osteoclast numbers in a rat model of osteoarthritis pain. Osteoarthritis. Cartilage..

[41] Cattaneo A (2010). Tanezumab, a recombinant humanized mAb against nerve growth factor for the treatment of acute and chronic pain. Curr.Opin.Mol.Ther.

[42] Balanescu AR, Feist E, Wolfram G, Davignon I, Smith MD, Brown MT, West CR (2014). Efficacy and safety of tanezumab added on to diclofenac sustained release in patients with knee or hip osteoarthritis: a double-blind, placebo-controlled, parallel-group, multicentre phase III randomised clinical trial. Ann.Rheum.Dis.

